# Corprological and haematological parameters of albino mice (*Mus musculus*) concurrently infected with *Heligmosomoides bakeri* and *Trypanosoma brucei*

**Published:** 2013-09-13

**Authors:** A.I. Onyeabor, M.I. Wosu, C.C. Ohaeri

**Affiliations:** 1 *Department of Veterinary Microbiology and Parasitology, College of Veterinary Medicine, Micheal Okpara University of Agriculture, Umudike, Nigeria*; 2 *Department of Biological Sciences, College of Natural and Applied Sciences, Micheal Okpara University of Agriculture, Umudike, Nigeria*

**Keywords:** Albino mice, Concurrent infection, *Heligmosomoides bakeri*, *Trypanosoma brucei*

## Abstract

The effect of concurrent infection with *Trypanosoma brucei* (*T. brucei*) and *Heligmosomoides bakeri* (*H. bakeri*) was investigated in this study. Thirty adult male albino mice were used for the study. The mice were divided into six groups of five mice each. Group 1 served as uninfected control, Groups 2 and 3 were infected with *H. bakeri* and *T. brucei* respectively, Group 4 received both *T. brucei* and *H. bakeri* on the same day, Group 5 was experimentally infected with *H. bakeri* three days after *T. brucei* infection, while Group 6 was infected with *T. brucei* three days after *H. bakeri* infection. Blood and faecal samples were collected and analyzed weekly to determine the faecal egg counts (FEC), packed cell volume (PCV) and level of parasitaemia (LP). Weekly body weights (BW) were also recorded. FEC and parasitaemia increased in all the infected groups during the study, but these were significantly (*p*<0.05) higher in the multiple-infection (groups 4, 5 and 6) than those with the single infection (groups 2 and 3). The same trend was also observed in the BW and PCV (*p*<0.05). The level of infection produced by single infection with *T. brucei* and *H. bakeri* respectively were similar (*p*<0.05). All treatment groups were significantly (*p*<0.05) different from the control group. From the results, it was concluded that concurrent helminth and protozoan parasite infections produced more deleterious effect on the host when compared with single infection with either parasite. However, the pathology produced by concurrent infection was more severe when the host was exposed to the protozoan parasite before the helminth parasite.

## Introduction

Gastrointestinal helminthosis is a major militating factor against profitable animal production around the world (Fabiyi, 1979; Chiejina, 1986). The prevalence and severity of gastrointestinal helminth infections have also continued to increase. This could be attributed to an increase in the occurrence of multiple infections involving these helminths and other pathogens in affected flock (Griffin *et al.*, 1981; Goosens *et al.*, 1997). Concurrent infections involving gastrointestinal nematodes and *Trypanosoma* species are of particular interest as a result of the reported increase in occurrence of mixed infections, especially in trypanosome endemic regions of Africa (Darji *et al.*, 1992). Grazing animals are usually exposed to concurrent infections and the presence of one parasite may affect other parasites within the host system (Nwosu *et al.*, 2006). The present study was therefore designed to further elucidate the pathologic effects produced by mixed infections using *Trypanosoma brucei* (*T. brucei*) and *Heligmosomoides bakeri* (*H. bakeri*). Such knowledge will have a positive inference for increasing profitability of livestock production ventures in parasite endemic areas.

## Material and Methods

### Experimental animals

Thirty adult inbred male albino mice (*Mus musculus*) weighing between 28-30 g were purchased from the Faculty of Veterinary Medicine, University of Nigeria, Nsukka, Nigeria. They were kept in rat cages with feed (Vital Feed, Nigeria) and water provided *ad libitum*. The experimental procedureswere approved by the Ethical committee of Micheal Okpara University of Agriculture, Umudike, Nigeria. The National Institute of Health Principles of Laboratory Animal Care (NRC, 1985) were observed.

### Sources of parasites

The *H. bakeri* used in the experiment was obtained from the Department of Veterinary Parasitology and Entomology, University of Nigeria, Nsukka. The parasites were passaged and maintained in mice. Faecal material obtained from the mice were collected, lightly macerated and centrifuged at 313 xg for 2 minutes.

The sediment obtained was reconstituted into a paste and cultured for 10 days at 25ºC. Infective larvae (L_3_) of *H. bakeri* were harvested using the modified Baermann technique (Hansen and Perry, 1994). The *T. brucei* used in the experiment was also obtained from the Department of Veterinary Parasitology and Entomology, University of Nigeria, Nsukka. The parasites were maintained in mice.

### Experimental design

The animals were randomly placed in six groups of five animals each and acclimatized for two weeks prior to the start of the experiment. Group 1 served as the uninfected control group, Groups 2 and 3 were infected independently with *H. bakeri* infective larvae (L_3_) and *T. brucei*, respectively. Group 4 was infected with both *T. brucei* and *H. bakeri* L_3_ on the same day. Group 5 was infected with *T. brucei* first and after three days with *H. bakeri* infective larvae while Group 6 received *H. bakeri* infective L_3_ followed by *T. brucei* three days later.

Individual body weights and packed cell volume (PCV) were recorded before the commencement of the experiment and every week subsequently till the end of the experiment. Individual FEC and parasitaemia were also determined and recorded every week from Week 1 post infection till the end of the experiment. The experiment lasted for 10 weeks.

### Infection of the mice

### H. bakeri

The mice were infected orally with 150 *H. bakeri* L_3_ suspended in 200µl of distilled water. The mice were properly restrained before dosing with L_3_ and exact volume of the larval suspension were delivered with an automatic micropipette (Finnipipette®; Labsystems Oy, Helsinski, Finland), adapted to take a blunt, slightly curved 18-guage needle as dosing aid (Fakae, 2001).

### T. brucei

The mice were inoculated intraperitoneally with 0.2 ml of infected blood containing approximately 1.0 × 10^5^ *T. brucei*/ml.

### Faecal egg counts

Weekly faecal egg counts (FEC) determination was carried out on the animals in all experimental groups using the salt floatation method and modified McMaster technique as egg counts increased as described by MAFF (1977).

### Determination of the level of parasitaemia

The patency of *T. brucei* infection was determined by wet film examination of blood from a tail snip by the method of Murray *et al*. (1983). Parasitaemia was estimated using the rapid matching technique as described by Herbert and Lumsden (1976).

### PCV

The PCV was determined by the Microheamatocrit method. The mice were bled from the tail directly into heparinized capillary tubes.

### Body weight determination

The mice were weighed using a desktop balance (Sartorius GMBH Gottingen Germany).

### Data analysis

Data obtained were summarized as means ± standard errors and the differences between means determined at the 5% level of significance using Analysis of Variance (ANOVA).

## Results

**Fig. 1:**
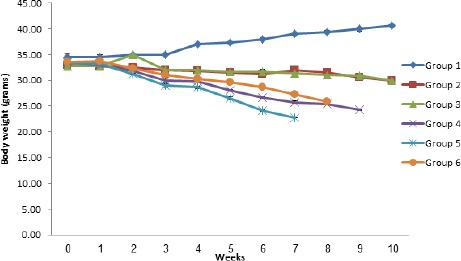
Mean body weights of albino mice experimentally infected singly or concurrently with *H. bakeri* and *T. brucei* and their control.

The effect of concurrent infection with *T. brucei* and *H. bakeri* on body weight is shown in figure 1.

**Fig. 2:**
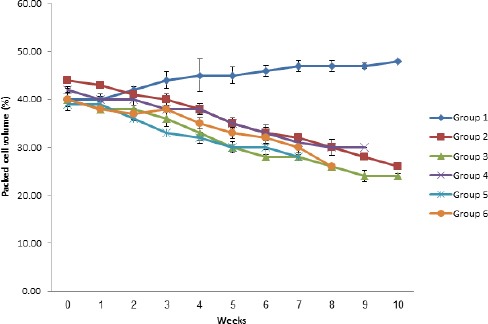
PCV of albino mice experimentally infected with *T. brucei* and *H. bakeri* and their control.

By the second week post infection, there was a significant (*p<*0.05) decrease in mean body weight in all treated groups when compared with the control group (Group 1). This trend continued till week 10 with the control group showing significant (*p<*0.05) increase in weight when compared with all infected groups. The decrease in mean body weights observed was also significantly (*p<*0.05) higher in the multiple infection groups (Groups 4, 5 and 6) when compared with the single infection groups (2 and 3), with Group 5 showing a more marked (*p<*0.05) decrease than all the groups. There was a marked (*p<*0.05) drop in PCV of all infected groups (Fig. 2).

**Fig. 3:**
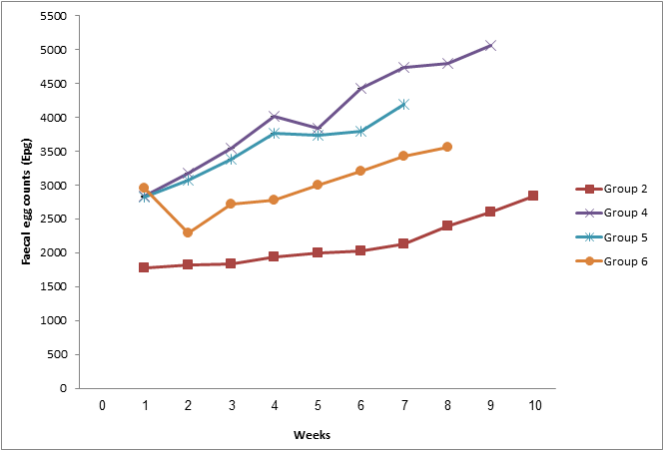
Egg counts of mice experimentally infected with *H. bakeri* alone or concurrently with *T. brucei* and their control.

Mortalities were recorded by the 7^th^, 8^th^ and 9^th^ weeks for the multiple infection groups (groups 5, 6 and 4) with PCV values of 28±0.58%, 26±0.58% and 30±0.00% respectively. Animals in Groups 2 and 3 survived to the end of the study (week 10) although there was a significant (*p<*0.05) decrease in PCV when compared with the uninfected (Group 1). The *H. bakeri* infection became patent between days 6 and 7 for Group 2 while for the multiple infection groups, patency was observed between days 2 and 4 post infection. Group 5, however, showed an earlier patency (day 2). Following patency, FEC continued to rise progressively in all infected groups until the end of the study (Fig. 3).

**Fig. 4:**
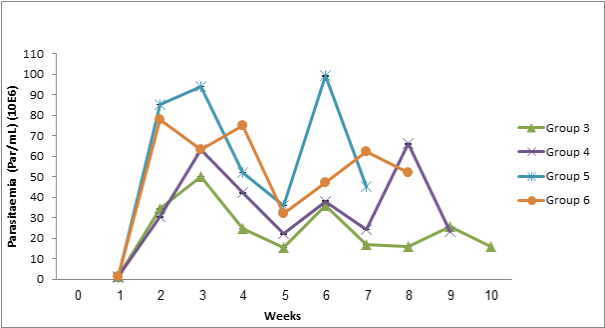
Parasitaemia of mice experimentally infected with *T. brucei* alone or concurrently with *H. bakeri* and their control.

However, Groups 4, 5 and 6 infected concurrently with *T. brucei* had significantly (*p<*0.05) higher egg counts than Group 2 that was infected with only *H. bakeri*. The effect of infection on parasitaemia is shown in figure 4. The prepatent period of *T. brucei* infection was 2-3 days. Among the concurrently infected groups, parasitaemia was highest in Group 5.

## Discussion

Progressive drop in weight could be attributed to the observed manifestations of typical signs of disease such as reduced food and water intake, reduced activity, sleepiness, low PCV and ultimately, death. In this study, the severity of weight loss was more marked in groups concurrently infected with *T. brucei* and *H. bakeri* than in the single infection groups. This agrees with the findings of Faye *et al*. (2002) and Kaufmann *et al*. (1992).

The shorter pre-patent periods observed in the concurrently infected groups may have occurred due to the additive effects of both parasites in the host, where the presence of one parasite results in a more favorable host environment for the proliferation of the second parasite. This could also be attributed to the suppression of the immune response of the host due to the earlier introduction of the first parasite (Nwosu *et al.*, 2001). This was demonstrated specifically in the group which received *T. brucei* earlier than *H. bakeri*. Trypanosomes have been reported to compromise the immune system of affected hosts (Albright *et al.*, 1978; Van Dam *et al.*, 1981).

This suppression in immunity could have led to increased pathological effects observed in this group. Anaemia was also observed in all the infected groups in the study.

There was a drastic reduction in the PCV of both single and multiple infection groups as both the levels of parasitaemia and FEC increased. Anaemia is a predominant symptom and a reliable indicator for the severity of trypanosome infections (Anosa, 1988). Anaemia is also a major finding in gastrointestinal nematode infections (Steel *et al.*, 1982; Behrens *et al.*, 2001).

The anaemia in mice was manifested by varying degrees of reduction beyond pre-infection values of the PCV. The concurrently infected groups which received *T. brucei* before *H. bakeri* had an earlier onset and a more severe anaemia, followed respectively by those which received *H. bakeri* before *T. brucei*; those which received *T. brucei* and *H. bakeri* on the same day; those infected singly with *T. brucei* and those singly infected with only *H. bakeri*. This also agrees with the findings of Mbaya *et al*. (2009) in gazelles concurrently infected with *Haemonchus contortus* and *T. brucei*.

They concluded that concurrent infection produced more depressing effects on all blood parameters when compared with single infection groups. Similar findings have also been reported by Nwosu and Ikeme (1992) in *T. brucei* infection in dogs and by Udensi and Fagbenro-Beyioku (2012) in mice.

The intensity of the anaemia in the concurrently infected groups may have resulted from a synergistic action of cell injury caused by trypanosomosis (Igbokwe, 1994) and haematophagous activity of *H. bakeri* (Fabiyi, 1987) leading to a high rate of red cell loss.

Therefore, the high rate of red cell loss may be due to the combined effects of a haemorrhagic and haemolytic anaemia related to the presence of both parasites in the host (Dargie and Allonby, 1979). It is noteworthy that the response of the group which received both infections on the same day for both parameters (PCV and FEC) was similar to the effect produced by a single infection with the individual parasites. The results also imply that *T. brucei* infection superimposed on *H. bakeri* infection aggravated the damage caused by the helminth parasite. This agrees with the findings of other researchers (Philips *et al.*, 1974; Fakae *et al.*, 1994; Onah and Wakelin, 1999; Chiejina *et al.*, 2005). Also, mortality rates were higher in all groups exposed to multiple rather than single infections.

In conclusion, the results showed that concurrent helminth and protozoan infections produced more pathologic effects than single infection with individual parasites. However, the severity of infection increased when the animals were exposed first to the protozoan parasite prior to the helminth parasite as seen by earlier onset and more acute progress of disease. It is therefore recommended that in trypanosome endemic areas, routine screening and prophylaxis for both parasites should be carried out for more effective management and disease control.
